# Clinical efficacy of open palmar approach combined with distal radial cancellous bone transplantation and internal fixation in the treatment of scaphoid nonunion

**DOI:** 10.3389/fsurg.2024.1372588

**Published:** 2024-11-15

**Authors:** Gang Li, Rui Li, Jafeng Long, Xuehai Ou, Shaoyan Shi

**Affiliations:** Department of Hand Surgery, Honghui Hospital, Xi'an Jiaotong University, Xi’an, China

**Keywords:** open palmar approach, scaphoid nonunion, distal radial cancellous bone, bone graft, internal fixation

## Abstract

**Background:**

To investigate the clinical efficacy of the open palmar approach combined with distal radial cancellous bone transplantation and internal fixation in the treatment of scaphoid nonunion.

**Methods:**

From March 2019 to March 2022, our center conducted a clinical observation on 19 patients with scaphoid nonunion, including 14 males and 5 females, aged 18–63 years, with an average age of (43.5 ± 15.5) years. The surgical approach involved open palmar access combined with distal radial cancellous bone transplantation and internal fixation, followed by three months of immobilization with a splint. Postoperative x-ray examinations were performed at 6 months to observe bone graft healing and functional recovery.

**Results:**

Follow-up of the 19 patients ranged from 6 to 24 months, with an average of 18 months. All patients achieved bony union, with an average healing time of 6 months. Postoperatively, wrist joint function was assessed using the modified Mayo wrist joint function scoring criteria: excellent in 15 cases, good in 2 cases; an excellent and good rate of 89.5%.

**Conclusion:**

The open palmar approach combined with distal radial cancellous bone transplantation and internal fixation is effective in treating scaphoid nonunion. The procedure is minimally invasive, allowing complete exposure of the fractured end of the scaphoid and the distal radius through the same incision. It facilitates easy cleaning of the fracture end, as well as convenient bone harvesting, grafting, and internal fixation. The postoperative efficacy is reliable, demonstrating significant advantages in the treatment of scaphoid nonunion.

## Introduction

Scaphoid nonunion, a challenging clinical entity, poses significant functional impairments and discomfort for patients (1, 2). The condition often manifests with symptoms such as pain, reduced grip strength, and restricted wrist joint mobility (3). In the absence of appropriate intervention, scaphoid nonunion can lead to long-term complications, impacting the overall quality of life for affected individuals (4, 5). Moreover, scaphoid nonunion is commonly associated with complications like humpback deformity and dorsal intercalated segment instability, which may necessitate additional corrective surgical measures to restore proper carpal alignment (6).

The prevalence of scaphoid nonunion varies but is notably higher in individuals with delayed diagnosis or insufficient initial treatment, complicating the path to recovery (3). The challenges in treating scaphoid nonunion arise due to the scaphoid's unique anatomy, its tenuous blood supply, and the variable presentation of fractures, which often lead to healing complications. In recent years, there has been a growing interest in exploring innovative surgical approaches to address scaphoid nonunion effectively. However, selecting a surgical strategy is complicated due to diverse patient presentations, nonunion locations, types, and bone graft sources (7, 8). A variety of bone grafting techniques, such as iliac crest bone grafts, vascularized bone grafts from the radius or second metacarpal, and modified butterfly grafts, have been explored to achieve union in scaphoid nonunion cases (1, 9–11). In a previous study, Li et al. achieved successful outcomes in 12 patients with stages I–III scaphoid nonunion advanced collapse using a closing-wedge distal radius osteotomy and vascularized bone graft, resulting in union, improved function, pain relief, and preserved wrist function over a 2–11 year follow-up (12). Recently, the open palmar technique combined with distal radial cancellous bone transplantation and internal fixation has gained great attention. This method aims to provide a minimally invasive solution with enhanced exposure of the scaphoid fracture site, facilitating thorough cleaning, bone grafting, and secure internal fixation (13). For instance, Burnier et al. demonstrated a 93.5% healing rate in 77 patients using an arthroscopic cancellous bone graft approach for scaphoid nonunion, showcasing outcomes comparable to or even surpassing those of pedicled vascularized grafts (14).

Despite these advances, significant gaps remain in the current literature, particularly regarding minimally invasive techniques and long-term functional outcomes following different surgical approaches (5). The open palmar approach, combined with distal radial cancellous bone transplantation and internal fixation, has recently gained attention for its potential to offer a less invasive solution with improved exposure of the fracture site, enabling thorough cleaning, grafting, and stable fixation. However, this method requires further evaluation to confirm its efficacy and role in the surgical treatment of scaphoid nonunion (15, 16). The objective of this study is to investigate the clinical efficacy of the open palmar approach combined with distal radial cancellous bone transplantation and internal fixation in treating scaphoid nonunion. By evaluating a cohort of 19 patients over a period of 6–24 months, we aim to assess postoperative outcomes, including fracture healing, functional recovery, and complications. This research contributes to the ongoing exploration of optimal surgical strategies for scaphoid nonunion, emphasizing the importance of achieving bony union, restoring wrist joint function, and enhancing overall patient well-being.

## Materials and methods

Ethic approval for this study (No. 2021097522) was approved by the Ethical Committee of Honghui Hospital, Xi'an Jiaotong University, Xi'an, China.

A total of 19 patients were included in the study, comprising 14 males and 5 females, with ages ranging from 18 to 63 years. Among the cases, 14 involved the left hand and 5 the right hand. There were 12 cases of waist fractures and 7 cases of distal fractures. The duration from injury to hospital admission ranged from 8 weeks to 3 years, with an average of 19 months. Inclusion criteria consisted of scaphoid nonunion diagnosed via x-ray and/or CT scan, with a history of scaphoid fracture and a minimum of 8 weeks post-injury. Patients presenting with other upper extremity fractures, vascular diseases, or chronic inflammatory conditions were excluded.

Clinical manifestations in 17 patients included varying degrees of pain during wrist joint movement, tenderness at the snuffbox, reduced grip strength, and restricted wrist joint flexion, extension, and radial deviation. Two asymptomatic patients (aged 58 and 60) were incidentally found to have scaphoid nonunion after palm bone fractures, with a significant decrease in grip strength attributed to long-term soft tissue damage. X-ray and CT examinations revealed varying degrees of bone sclerosis, absorption, and cystic changes at the fractured ends of the scaphoid in all patients. Among the 19 patients, 11 had not received any treatment, 6 underwent plaster fixation, and 2 had previously undergone closed reduction with Kirschner wire fixation.

### Surgical procedure

After brachial plexus nerve block anesthesia, the patient was placed in a supine position with the injured limb abducted. A routine disinfection and draping were performed, and a tourniquet was applied. The surgical approach involved an “S-shaped” incision, approximately 6 cm in length, extending from the radial styloid process to the scaphoid tubercle. The palmar branch of the radial artery was dissected and protected, and the radial artery palmar skin branch was ligated. A retractor was used to expose the scaphoid tubercle, the radio scaphoid ligament, and the pronator quadratus muscle. The radio scaphoid ligament was incised longitudinally. Two 1.0 mm Kirschner wires were drilled into the far and near poles of the scaphoid to pull apart the fractured ends. The fractured ends were cleaned, and fresh bone surfaces were exposed. A rectangular bone groove was created using a oscillating saw to receive the bone flap. The volume of the defect was measured. Cancellous bone from the radial bone distal end was harvested and placed in the scaphoid defect. The defect was filled, and the Kirschner wires were removed. A hollow core compression screw was inserted into the bone groove to fix the fractured ends. The wound was irrigated, and the extensor retinaculum and joint capsule were repaired. The wound was closed layer by layer, and a sterile dressing was applied.

### Postoperative management

The limb was elevated within 24 h postoperatively, and local cold compress was applied. Dressing changes were performed on the second day, and x-ray examination of the scaphoid was conducted. The forearm brace was continued for two weeks, and stitches were removed after two weeks. Subcutaneous Kirschner wires were removed after four weeks if x-ray examination indicated satisfactory fracture alignment. Postoperative x-ray evaluations were regularly performed to assess fracture healing. Weight-bearing and functional exercises were initiated gradually after brace removal. The hollow core compression screw could be left in place and did not need to be removed.

### Postoperative follow-up and functional assessment

Regular follow-up was conducted for 19 patients, including 14 clinic visits and 5 through online. Healing of the fracture, wrist joint pain, and functional recovery were assessed. Regular x-ray examinations were performed to evaluate scaphoid healing. Pain was assessed using the Visual Analogue Scale (VAS), and wrist joint function was assessed using the modified Mayo wrist joint score.

### Typical case 1

**Figure 1:**
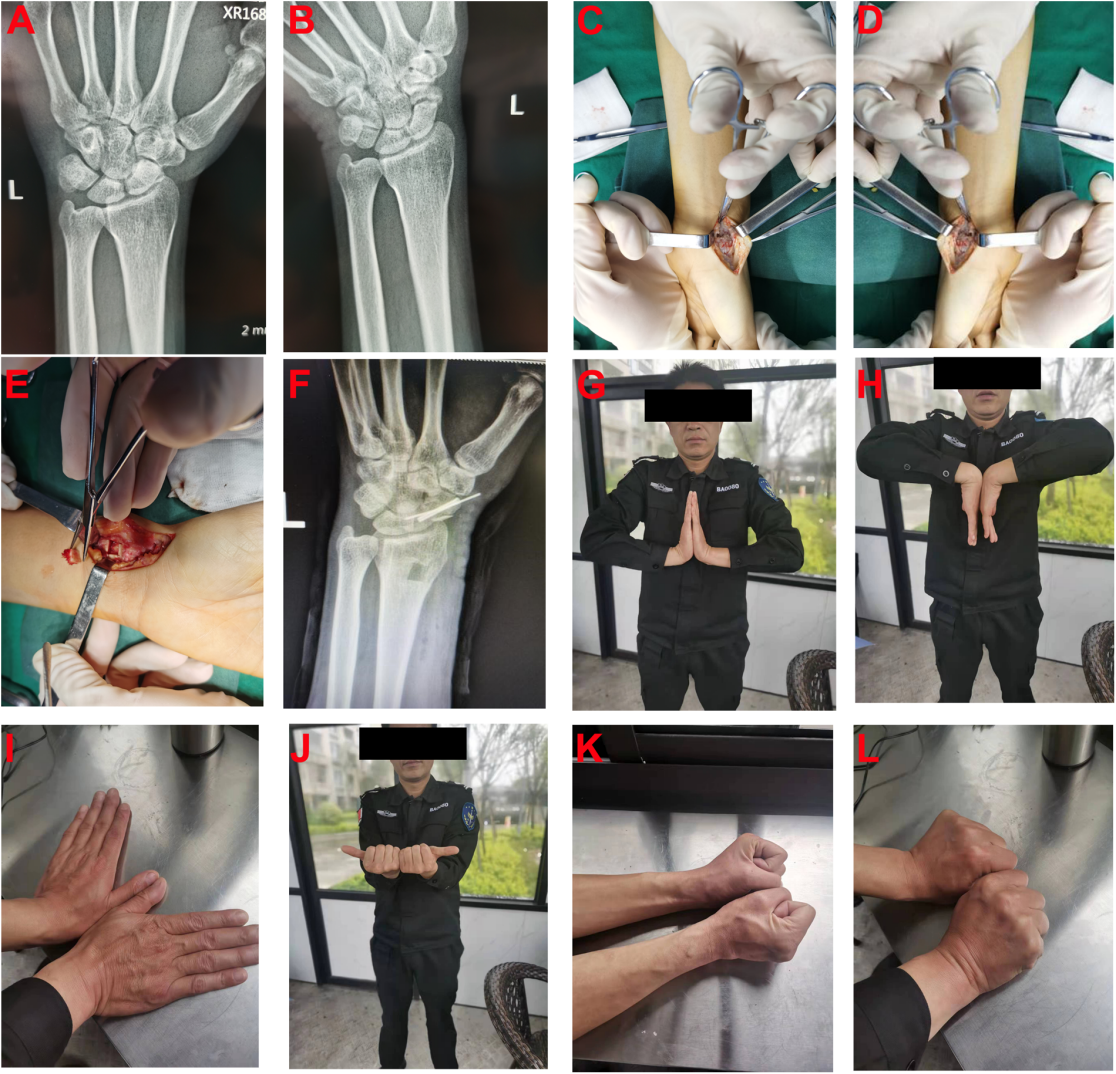
A 49-year-old male patient who underwent open palmar approach combined with distal radial cancellous bone transplantation and internal fixation. **(A,B)** Preoperative Anteroposterior Image and scaphoid positioning Image; **(C)** An “S-shaped” Surgical Incision; **(D)** Cleaning of scaphoid fracture ends scars and sclerotic bone; **(E)** Retrieval of radial bone distal block; **(F)** Postoperative scaphoid positioning Image; **(G–L)** Activity of wrist after 3 months follow up.

The patient is a 49-year-old male farmer who was admitted to our department due to left wrist pain and limited mobility for 2 years. Two years ago, he experienced a fall while working, resulting in swelling and pain in the left wrist, along with restricted movement. Without seeking timely medical attention, he continued to engage in strenuous physical labor. Due to a subsequent decline in grip strength in his left hand, affecting his work, he sought medical attention at our hospital. Upon admission, the patient's overall condition was good. Specialized examination revealed no apparent swelling in the left wrist, normal skin temperature, positive tenderness at the snuffbox, limited active flexion and extension, and restricted radial deviation in the left wrist. X-ray examination revealed a continuous interruption of the distal scaphoid bone in the left wrist, indicative of scaphoid nonunion. The fracture ends showed visible white sclerotic bone changes. After comprehensive preoperative examinations, it was decided to perform an open palmar approach combined with distal radial cancellous bone transplantation and internal fixation under brachial plexus anesthesia, considering the patient's condition (Figure 1).

### Typical case 2

**Figure 2:**
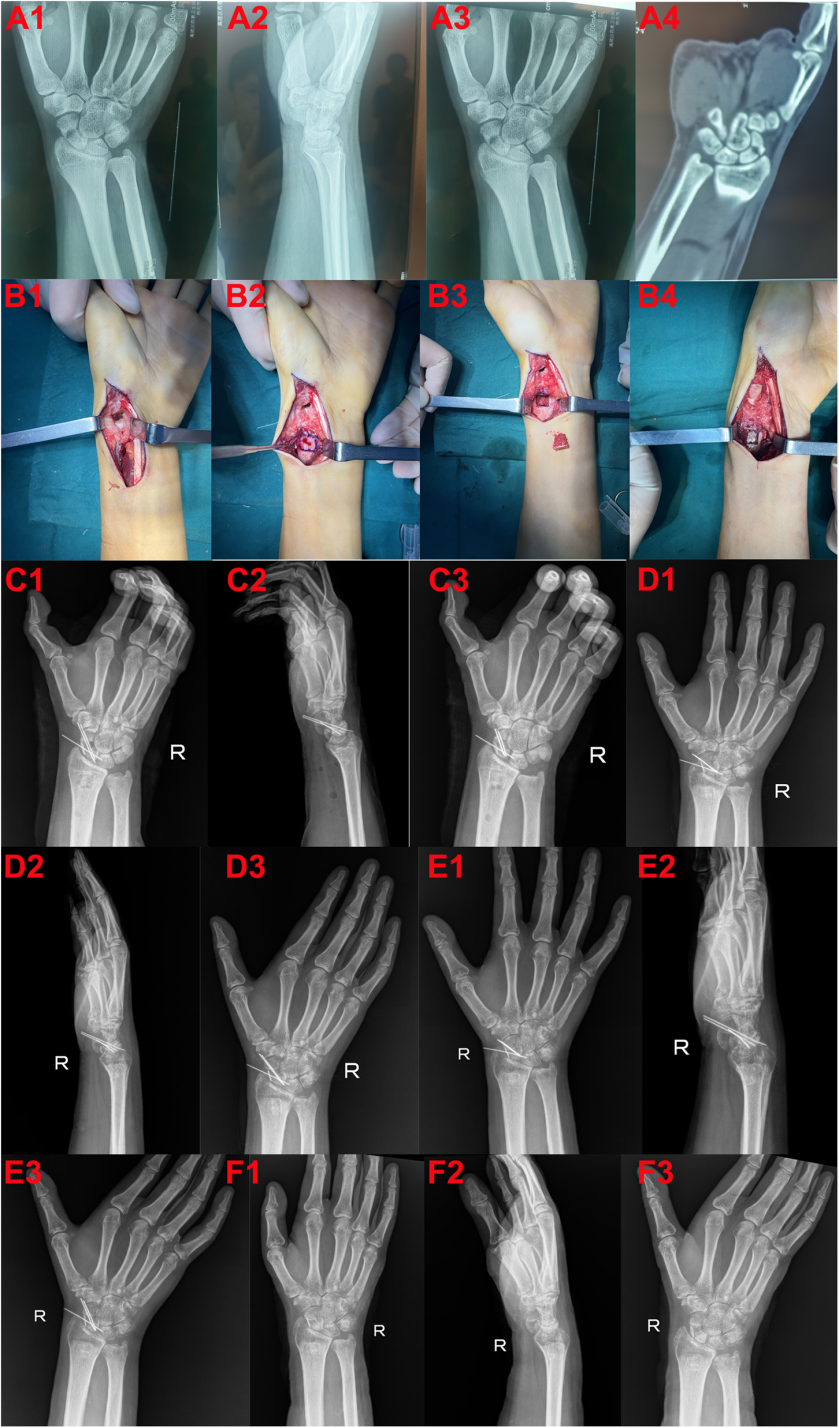
A 27-year-old male patient who underwent surgery. **(A1–3)** Preoperative radiographs of the wrist in anteroposterior, lateral, and ulnar deviation views. **(A4)** Preoperative CT scan showing scaphoid nonunion. **(B1)** Intraoperative image depicting debridement of the scaphoid fracture site. **(B2)** Location of the distal radius where autologous bone graft was harvested. **(B3)** Harvested autologous bone from the distal radius. **(B4)** Implantation of autologous bone graft into the scaphoid nonunion site. **(C1–3)** Postoperative radiographs of the wrist in anteroposterior, lateral, and ulnar deviation views. **(D1–3)** Radiographs of the wrist one month postoperatively in anteroposterior, lateral, and ulnar deviation views. (**E1–3)** Radiographs of the wrist two months postoperatively in anteroposterior, lateral, and ulnar deviation views. **(F1–3)** Radiographs following removal of internal fixation, showing anteroposterior, lateral, and ulnar deviation views.

The patient is a 27-year-old male who sustained an injury after slipping and falling while riding a motorcycle on his way to work, landing on his right upper limb and shoulder. Following the accident, the patient experienced significant pain and limited mobility in the right wrist. X-rays revealed fractures of the right scaphoid and radial styloid. Based on the severity of the fractures, surgical intervention was recommended. The patient subsequently underwent an open palmar approach combined with distal radial cancellous bone transplantation and internal fixation. At the two-month follow-up, x-ray imaging revealed satisfactory healing of the scaphoid fracture with evidence of bone consolidation at the graft site. The internal fixation hardware was successfully removed, and the patient demonstrated excellent postoperative recovery with restored wrist mobility and no significant pain or complications (Figure 2).

### Statistical analysis

Statistical analysis was performed using SPSS 23.0 software. Descriptive statistics were presented as mean ± standard deviation (Mean ± SD). Group comparisons were made using *t*-tests, with *P* < 0.05 considered statistically significant.

## Results

Postoperative incision healing was excellent, all classified as Grade I healing, and sutures were removed on schedule. X-ray examinations revealed anatomical reduction of the fracture ends, with screws inserted centrally along the longitudinal axis of the scaphoid. There was no screw penetration. The follow-up period ranged from 6 to 24 months. Guided by the fracture healing status, functional exercises were initiated, and all patients achieved bony union, with an average healing time of 12.5 (range: 10–16) weeks.

No complications such as wound infection, avascular necrosis of the scaphoid, nonunion of bone grafts, arthritis, or screw loosening or breakage were observed during the follow-up. The last follow-up showed significant improvement in VAS scores and Mayo scores compared to preoperative scores. Grip strength, palmar flexion, and dorsal extension of the wrist significantly increased. The outcomes were excellent in 15 cases, good in 2 cases, with an excellent and good rate of 89.5%. All differences were statistically significant (*P* < 0.05, Table 1).

**Table 1:** Intraoperative and postoperative final follow-up functional comparisons (mean ± SD).

Items	Preoperative	Postoperative final followed-up	*P*-value
Activity of wrist (°)			
Palm flexion	45 ± 17	65 ± 14	0.04
Dorsal extension	39 ± 18	59 ± 20	0.03
Pronation	76 ± 10	77 ± 11	0.16
Supination	78 ± 11	80 ± l2	0.08
Strength of hand grip (kg)	16.9 ± 4.0	31.8 ± 3.7	0.02
Improved Mayo score	46.3 ± 11.7	69.8 ± 10.5	0.01
VAS score	3.4 ± 1.6	1.0 ± 1.0	0.03

## Discussion

The present study focused on the clinical efficacy of the open palmar approach combined with distal radial cancellous bone transplantation and internal fixation in the treatment of scaphoid nonunion. The encouraging outcomes observed in our cohort shed light on the potential of this surgical technique as a viable intervention for scaphoid nonunion.

The average healing time of 12.5 weeks in our study aligns with the findings of previous research investigating various surgical interventions for scaphoid nonunion (17). For instance, Hegazy et al. reported comparable outcomes with a mean healing time of 14 weeks using bone grafting combined with internal fixation (18). The relatively short healing period in our cohort suggests that the open palmar approach, in conjunction with distal radial cancellous bone transplantation, facilitates robust and timely bony union. Furthermore, our strict postoperative anti-smoking education also prevented nonunion of fractures. This is consistent with the results reported by Fan et al., which emphasized the importance of stringent postoperative care and lifestyle modifications, such as smoking cessation, in achieving successful outcomes (19). Consequently, our comprehensive approach, incorporating both surgical techniques and postoperative smoking cessation education, contributes to favorable outcomes in terms of fracture healing.

The absence of complications such as wound infection, avascular necrosis, or graft nonunion during our follow-up period underscores the safety and reliability of the described surgical technique. This aligns with the findings of studies advocating for the benefits of incorporating cancellous bone grafts in scaphoid nonunion surgeries (20). Despite advanced fixation techniques, management of scaphoid nonunion still need utilizing nonvascularized bone grafts, such as corticocancellous, cancellous, and strut grafts, to achieve union in waist and proximal pole fractures with good success rates (21). For instance, Bae et al. reported that the use of cancellous bone graft from the distal radius in conjunction with headless screw fixation for treating unstable scaphoid waist nonunion demonstrated a significant positive effect, achieving a 93.5% union rate (22). Comparing our findings to those of Kirschner wire vs. Herbert screw fixation for treating unstable scaphoid waist fracture nonunions, the rate of scaphoid union was similar, with Kirschner wire achieving a union rate of 91% (23). While the Kirschner wire technique was found to have shorter operative times and lower costs, our method's emphasis on headless screw fixation may offer advantages in terms of maintaining scaphoid anatomy and minimizing complications related to graft harvesting (11). Mechanically, the cancellous bone grafts contribute to osteogenic potential, aiding in the acceleration of fracture healing and minimizing the risk of nonunion.

Functional outcomes, as assessed by VAS scores and Mayo wrist joint scores, exhibited significant improvement postoperatively. The enhancement in grip strength and increased range of motion in wrist flexion and extension align with the findings of similar studies utilizing different surgical approaches (23, 24). The overall excellent and good rate of 89.5% in our cohort further emphasizes the clinical success of the open palmar approach combined with distal radial cancellous bone transplantation and internal fixation.

An important addition to our surgical planning was the use of preoperative CT imaging, which allowed for a more detailed evaluation of fracture characteristics. This preoperative imaging played a pivotal role in informing the surgical strategy, especially in cases where deformities were identified (25). Previous studies have indicated that preoperative recognition of such deformities can help guide intraoperative correction and ensure optimal fracture alignment (26). In our cohort, none of the cases required additional carpal realignment procedures, which may have contributed to the high union rates and functional recovery observed. Postoperative imaging protocols were another key factor in monitoring fracture healing and detecting early signs of complications. In cases where x-ray findings were inconclusive, CT scans provided a more definitive assessment of bony union, particularly in challenging cases of proximal pole fractures (27, 28). Incorporating advanced imaging techniques into the follow-up regimen allowed for more accurate and timely decisions regarding the removal of Kirschner wires and the initiation of weight-bearing exercises. Studies have emphasized the value of CT scans in confirming union, particularly in scaphoid fractures where plain radiographs may underestimate healing (29).

Despite the promising results, certain limitations should be acknowledged. The relatively small sample size and the absence of a comparative group limit the generalizability of our findings. Additionally, our follow-up period, while sufficient to assess short- to medium-term outcomes, may not capture late-onset complications or long-term functional deficits. Future studies should aim to include a larger cohort with a comparative control group, as well as longer-term follow-up to evaluate the sustained efficacy of the technique and potential complications such as hardware failure or osteoarthritis. Furthermore, the role of advanced imaging modalities, such as CT or MRI, in both preoperative planning and postoperative evaluation warrants further exploration in scaphoid nonunion management.

In conclusion, our study contributes to the growing body of evidence supporting the effectiveness of the open palmar approach combined with distal radial cancellous bone transplantation and internal fixation in treating scaphoid nonunion. The positive outcomes, in terms of timely healing, improved functional scores, and low complication rates, advocate for the consideration of this surgical technique in the management of scaphoid nonunion. Further multi-center, randomized controlled trials with extended follow-up durations are warranted to validate our findings and establish this approach as a gold standard in scaphoid nonunion management.

## Data Availability

The original contributions presented in the study are included in the article/Supplementary Material, further inquiries can be directed to the corresponding author.

## References

[1] Karaismailoglu B Fatih Guven M Erenler M Botanlioglu H. The use of pedicled vascularized bone grafts in the treatment of scaphoid nonunion: clinical results, graft options and indications. EFORT Open Rev. (2020) 5:1–8. 10.1302/2058-5241.5.190021 32071768 PMC7017592

[2] Testa G Lucenti L D'Amato S Sorrentino M Cosentino P Vescio A Comparison between vascular and non-vascular bone grafting in scaphoid nonunion: a systematic review. J Clin Med. (2022) 11:3402. 10.3390/jcm11123402 35743472 PMC9225170

[3] Yin Y Xu K Zhang N Yi Z Liu B Chen S. Clinical and epidemiological features of scaphoid fracture nonunion: a hospital-based study in Beijing, China. Orthop Surg. (2022) 14:2455–61. 10.1111/os.13478 36000517 PMC9531078

[4] Higgins JP Giladi AM. Scaphoid nonunion vascularized bone grafting in 2021: is avascular necrosis the sole determinant? J Hand Surg Am. (2021) 46:801–6.e802. 10.1016/j.jhsa.2021.05.014 34183203

[5] Gray RRL Halpern AL King SR Anderson JE. Scaphoid fracture and nonunion: new directions. J Hand Surg Eur Vol. (2023) 48:4S–10. 10.1177/17531934231165419 37704024

[6] Shin JW Kim DW Kwak DH Park JW Lee JI. A comparative study of volar locking-plate fixation with corticocancellous and pure cancellous bone grafts for scaphoid nonunion with dorsal intercalated segmental instability secondary to scaphoid humpback deformity. Injury. (2024) 55:111583. 10.1016/j.injury.2024.111583 38692209

[7] Van Nest D Ilyas AM. Scaphoid nonunion: a review of surgical strategies. Orthopedics. (2022) 45:e235–42. 10.3928/01477447-20220608-03 35700430

[8] Rogers MJ Ohlsen SM Huang JI. Fixation techniques for scaphoid nonunion. J Am Acad Orthop Surg. (2023) 31:783–92. 10.5435/JAAOS-D-23-00287 37307573

[9] Schott T Eisenberg KA Vuillermin CB Bae DS Waters PM Bauer AS. Donor-Site morbidity for iliac crest harvesting for pediatric scaphoid nonunion. J Hand Surg Am. (2023) 48:833.e1–833.e5. 10.1016/j.jhsa.2022.02.007 35513964

[10] Fujihara Y Yamamoto M Hidaka S Sakai A Hirata H. Vascularised versus non-vascularised bone graft for scaphoid nonunion: meta-analysis of randomised controlled trials and comparative studies. JPRAS Open. (2023) 35:76–88. 10.1016/j.jpra.2022.12.001 36793769 PMC9922807

[11] De Vitis R Passiatore M Perna A Tulli A Pagliei A Taccardo G. Modified Matti-Russe technique using a “butterfly bone graft” for treatment of scaphoid non-union. J Orthop. (2020) 19:63–6. 10.1016/j.jor.2019.11.030 32021039 PMC6994792

[12] Malizos KN Koutalos A Papatheodorou L Varitimidis S Kontogeorgakos V Dailiana Z. Vascularized bone grafting and distal radius osteotomy for scaphoid nonunion advanced collapse. J Hand Surg Am. (2014) 39:872–9. 10.1016/j.jhsa.2014.01.045 24656393

[13] Lin JS Grenier G Balch Samora J. Outcomes of pediatric scaphoid nonunions treated with distal radius cancellous autograft. J Pediatr Orthop. (2022) 42:260–4. 10.1097/BPO.0000000000002094 35153287

[14] Burnier M Loisel F Ardouin L Beauthier V Durand A Erhard L Treatment of scaphoid nonunion by arthroscopic cancellous bone grafting. Orthop Traumatol Surg Res. (2023) 109:103665. 10.1016/j.otsr.2023.103665 37499747

[15] Duncumb JW Robinson PG Williamson TR Murray IR Campbell D Molyneux SG Bone grafting for scaphoid nonunion surgery: a systematic review and meta-analysis. Bone Joint J. (2022) 104-B:549–58. 10.1302/0301-620X.104B5.BJJ-2021-1114.R1 35491585

[16] Schriever T Wilcke M. Residual flexion deformity after scaphoid nonunion surgery: 7-year follow-up study. J Hand Surg Eur Vol. (2023) 48:20–6. 10.1177/17531934221125355 36165430

[17] Shoji KE Simeone FJ Ozkan S Mudgal CS. Outcomes of local bone graft and fixation of proximal pole scaphoid nascent nonunions and nonunions. J Wrist Surg. (2020) 9:203–8. 10.1055/s-0040-1701512 32509423 PMC7263866

[18] Hegazy G Massoud AH Seddik M Abd-Elghany T Abdelaal M Saqr Y Structural versus nonstructural bone grafting for the treatment of unstable scaphoid waist nonunion without avascular necrosis: a randomized clinical trial. J Hand Surg Am. (2021) 46:462–70. 10.1016/j.jhsa.2021.01.027 33814250

[19] Fan S Suh N MacDermid JC Ross D Grewal R. Vascularized versus non-vascularized bone grafting for scaphoid nonunion without avascular necrosis: a randomized clinical trial. J Hand Surg Eur Vol. (2023) 48:648–53. 10.1177/17531934231158992 36861269

[20] Golubev I. Slight elongation of the scaphoid and cancellous bone graft without compression for treatment of scaphoid nonunions. Hand Clin. (2022) 38:351–6. 10.1016/j.hcl.2022.04.002 35985760

[21] Miller EA Huang JI. Traditional bone grafting in scaphoid nonunion. Hand Clin. (2024) 40:105–16. 10.1016/j.hcl.2023.08.001 37979982

[22] Bae JY Choi SW Lee W Song MG Song JS Kim JK. Cancellous bone graft from the distal radius and headless screw fixation for unstable scaphoid waist nonunion. Int Orthop. (2024) 48:487–93. 10.1007/s00264-023-05998-1 37796332

[23] Hegazy G Alshal E Abdelaal M Abdelaziz M Moawad M Saqr YM Kirschner wire versus herbert screw fixation for the treatment of unstable scaphoid waist fracture nonunion using corticocancellous iliac bone graft: randomized clinical trial. Int Orthop. (2020) 44:2385–93. 10.1007/s00264-020-04730-7 32683460

[24] Nakamoto JC Xavier RM Burgos FH Wataya EY do Carmo Iwase F Nakamoto HA Comparative analysis of scaphoid nonunion treatment with screw fixation and angular stable plate. Arch Orthop Trauma Surg. (2023) 143:2247–53. 10.1007/s00402-022-04625-9 36182974

[25] Konneker S Schachinger U Vogt PM. Magnesium-based compression screws in acute scaphoid fractures and nonunions. World J Surg. (2023) 47:1129–35. 10.1007/s00268-023-06937-2 36774451

[26] Miyamura S Shiode R Lans J Oka K Tanaka H Okada S Quantitative 3-D CT demonstrates distal row pronation and translation and radiolunate arthritis in the SNAC wrist. J Bone Joint Surg Am. (2023) 105:1329–37. 10.2106/JBJS.22.01350 37471563

[27] Bevers M Daniels AM van Rietbergen B Geusens P van Kuijk SMJ Sassen S Assessment of the healing of conservatively-treated scaphoid fractures using HR-pQCT. Bone. (2021) 153:116161. 10.1016/j.bone.2021.116161 34455117

[28] Saruhan S Savran A Yildiz M Sener M. Reconstruction of proximal pole scaphoid non-union with avascular necrosis using proximal hamate: a four-case series. Hand Surg Rehabil. (2021) 40:744–8. 10.1016/j.hansur.2021.07.003 34274497

[29] Lee GB Kim JK Shin YH. The effect of reformatting axis of computed tomography scans on the measurement of deformities in scaphoid waist nonunion. Orthop Traumatol Surg Res. (2021) 107:102980. 10.1016/j.otsr.2021.102980 34102335

